# Violence against children and intimate partner violence against women: overlap and common contributing factors among caregiver-adolescent dyads

**DOI:** 10.1186/s12889-019-8115-0

**Published:** 2020-01-29

**Authors:** Catherine Carlson, Sophie Namy, Andrea Norcini Pala, Milton L. Wainberg, Lori Michau, Janet Nakuti, Louise Knight, Elizabeth Allen, Carin Ikenberg, Dipak Naker, Karen Devries

**Affiliations:** 1 University of Alabama, School of Social Work, 3026 Little Hall, Box 870314, Tuscaloosa, AL 35487-0314 USA; 2 Raising Voices, Kampala, Uganda; 3 Columbia University, Social Intervention Group, New York, USA; 4 Columbia University, Department of Psychiatry, New York, USA; 5 London School of Hygiene and Tropical Medicine, London, UK

**Keywords:** Child maltreatment, Gender-based violence, Family, Adolescence, Africa

## Abstract

**Background:**

Intimate partner violence against women (IPV) and violence against children (VAC) are both global epidemics with long-term health consequences. The vast majority of research to date focuses on either IPV or VAC, however the intersections between these types of violence are a growing area of global attention. A significant need exists for empirical research on the overlap of IPV and VAC, especially in contexts with particularly high rates of both types of violence.

**Methods:**

This exploratory study includes secondary analysis of data from a cluster randomized controlled trial in Ugandan schools. Using baseline reports from a random sample of early adolescents attending school and their caregivers, this study uses a probability sample across all eligible schools of adolescent-caregiver dyads (*n* = 535). We categorized adolescent-caregiver dyads into four groups: those reporting VAC ‘only’, IPV ‘only’, both VAC and IPV, or ‘no violence’. Two separate multinomial logistic regression models for male and female caregivers explored adolescent and caregiver characteristics associated with the VAC ‘only’, the IPV ‘only’, or the both VAC and IPV dyads, each compared to the ‘no violence’ dyad.

**Results:**

One third of dyads reported both IPV and VAC and nearly 75% of dyads reported VAC or IPV. Dyads reporting IPV were more likely to also report VAC. Common contributing factors for female caregiver-adolescent dyads with both VAC and IPV include lower SES, less caregiver education, higher caregiver mental distress, more frequent caregiver alcohol use, and caregivers who report less emotional attachment to their intimate partner. Male caregiver-adolescent dyads with both VAC and IPV included caregivers with less emotional attachment to their intimate partner and more attitudes accepting VAC.

**Conclusions:**

Findings reveal a significant overlap of IPV and VAC and the importance for violence prevention and response programming to consider coordinated or integrated programming. Unique results for female and male caregivers highlight the importance of a gendered approach to addressing IPV and VAC intersections.

**Trial registration:**

The trial was registered at clinicaltrials.gov, NCT01678846, on September 5, 2012.

## Background

Intimate partner violence against women (IPV) and violence against children (VAC) by caregivers are each global epidemics [1, 2] with long-term health consequences [3, 4]. The intersection of IPV and VAC is a growing area of global attention, with calls to explore integrated programming [5–7]. Yet, the vast majority of research and practice have focused on women and children separately [5, 8], with few empirical studies on the connection or intersection of the two types of violence. Studies that do exist are primarily from high income countries and report that IPV and VAC often occur within the same families [9–23] and neighborhoods [24], increasing risk of negative health effects [4, 17, 25–30].

A lack of evidence exists on the overlap of IPV and VAC using data from low- and middle-income countries, where higher rates of poverty and differing gender structures may have unique effects on intersecting violence in families. A study using a non-probability sample from a South African hospital [31] chart files reported a 47% rate of co-occurring IPV and VAC. Studies from Nigeria and Northern Uganda found that mothers who reported IPV were significantly more likely to also report child cruelty [32] or aggressive parenting [33]. The etiology of both IPV and VAC can be understood within an ecological framework [34, 35], where factors across multiple spheres of life drive the co-occurrence of violence. At an interpersonal level, social information processing conceptualizes the use of both types of violence due to attitudes accepting the use of violence in close relationships [36]. Intersecting violence may also be considered from a social disorganization theory perspective, where concentrated neighborhood disadvantage contributes to the occurrence of both IPV and VAC [24]. Other recent studies on intersecting IPV and VAC drawn upon a feminist framework, which contextualizes family violence and attitudes accepting violence in personal relationships within broader gender and power hierarchies [37, 38]. A qualitative study on the intersections of IPV and VAC in Uganda explores the use of violence against both women and children as a mechanism of enforcing power hierarchies and gender/childhood norms within families [37]. In-depth interviews in South Africa, revealed that even mothers’ homicidal violence against their children frequently occurred within a context of the women’s long-term exposure to IPV [38]. Both of these studies reveal women’s use of violence against children as their exercise of power over children, when unable to express power over their male partners.

Despite qualitative evidence on intersecting violence against women and children from Africa, a lack of research exists estimating the rates of overlapping violence in families particularly child and caregiver reports. Research from the independent VAC and IPV literature report several common factors that increase the risk of both types of violence [39, 40], such as substance abuse, mental health problems, or women and child rights [16, 41–46]. Before the development of effective, integrated programming, there is a need to better understand the overlap of IPV and VAC and shared contributing factors.

## Methods

### Data source

This exploratory study aims to understand shared and distinct factors across caregiver-adolescent ‘dyads’ experiencing no violence, IPV ‘only’, VAC ‘only,’ and overlapping IPV and VAC. Secondary analyses are performed using data from the Good Schools Study [47, 48], a cluster randomized controlled trial conducted between 2012 and 2014 to test the impact of Good School Toolkit (GST; www.raisingvoices.org). The cross-sectional follow-up survey included a probability sample of primary school students and caregivers from 42 eligible schools in Luwero District (half of which—21—had received the 18 month GST intervention). Participants were selected in two stages. After excluding 97 schools with fewer than 40 Primary 5 students and 20 additional schools with ongoing interventions in progress, 42 schools (representing 80% of students in the district) were randomly selected from a list of the 151 remaining schools in the district. A maximum of 130 students from Primary 5, 6 and 7 were randomly selected from each school (response rate 92.3%). Any caregiver (primary or other) of all grade 7 students were also invited to complete a survey (response rate 66%). A total of 828 adolescent and caregiver dyads completed surveys. For the purposes of these analyses, caregivers who were not currently partnered were excluded (*n* = 287), as well as caregivers for whose sex was not recorded or able to determine (*n* = 6). After these exclusions, a total of 535 adolescent-caregiver dyads were included in the secondary analyses. All surveys were administered face-to-face in the school using tablet computers by trained interviewers between June and July 2014. Caregivers were informed about the study and could opt their adolescent out from participating, and informed written consent was obtained from all students and caregivers. Any disclosure of serious maltreatment were referred to local child protection services and all children were offered counselling regarding of what they disclosed. Women reporting experiencing IPV were offered information and support in accessing domestic violence services [49]. Ethical approval was obtained from the Ugandan National Council for Science and Technology (SS 2520) and the Ethics Committee of the London School of Hygiene and Tropical Medicine (#6183). The study followed international ethical guidelines for researching violence against women and children [50, 51], and a full discussion of the ethical protocol for the parent study is published elsewhere [49].

### Measures

Measures used for the study came from cross sectional surveys with both adolescents and caregivers. All instruments were translated into Luganda. Cognitive interviewing and pre-testing of measures were carried out prior to use [52]. Interviews were conducted in Luganda or English. Demographic questions captured from caregivers and adolescents included age and sex. Caregivers reports also included their education status, religious affiliation, number of years with their intimate partner, and if they currently lived together. Adolescents reported if they currently live with one, two, or no biological parents. Table 1 describes the variables and measures used for adolescents and caregivers.

**Table 1:** Caregiver and adolescent variables and measures

	Adolescent Report	Caregiver Report
Age	Years	Years
Sex	Girl, Boy	Man, Woman
Education		Primary vs. Secondary or more
Religious affiliation		Christian or Muslim
Relationship duration		Years with current partner
Living status	Lives with biological parent (Y/N)	Lives with current partner (Y/N)
Household size		Number of persons in household
SES		Index (See Attachment)
Physical Disability	Difficulty performing any basic universal tasks (Washington Group Short Set)	
Mental distress	Strengths and Difficulties Questionnaire (alpha = .82)	Self-Report Questionnaire (alpha = .92)
Alcohol Use		Alcohol use frequency in past 30 days
Relationship quality	Family connectedness (alpha = .72)	Emotional attachment *(*Relationship Structure Questionnaire*;* alpha = .90)
Attitudes towards violence		Attitudes against VAC (alpha = .82)
Violence		Emotional or physical violence against children (IPSCAN) Emotional, physical, or sexual violence (WHO Women’s Health and Life Events Questionnaire)
Intervention		Adolescent attends either an intervention or control school in the parent study

#### Socioeconomic status (SES)

Confirmatory factor analysis (CFA) was performed to test the 1-latent factor “SES” (items are listed in Appendix A). Weighted Least Square with adjusted Mean and Variance (WLSMV) estimation was used to analyze SES items, which consisted of binary or ordinal variables [53]. The model goodness-of-fit was evaluated with the Root Mean Square Error of Approximation (RMSEA) (≤.08 or .05), and Comparative Fit Index (CFI) and Tucker-Lewis index (TLI) (≥.95 or .90) [53–56]. The 1-factor model showed poor fit: RMSEA = .10 and CFI/TLI = .76/.71. The exclusion of items with non-significant loadings (*p* > .05) improved the fit of the model: RMSEA = .05, CFI/TLI .97/.96. The latent factor’s (standardized) score was exported and used as a measure of SES, a higher score indicates higher SES. Reliability for SES and other measures was assessed with Ordinal alpha, which outperforms Cronbach’s alpha when ordinal variables are analyzed [57]. R and *psych* package (Revelle, 2013) was used to compute Ordinal alpha; coefficients higher than 0.70 indicate good reliability.

#### Physical disability

Adolescents were asked questions on physical disability from the Washington Group short set. A dummy variable was created to measure if respondents reported any functional impairment, compared to no impairment.

#### Mental distress

The 20-item Self-Report Questionnaire (SRQ) [58] assessed caregiver’s symptoms of mental distress. Scores range from 0 to 20 with higher numbers indicating more distress (Ordinal Alpha = 0.9). The Strengths and Difficulties Questionnaire (SDQ) measured child reports of symptoms of common childhood and adolescent mental disorders, including depression, anxiety, and conduct disorder [59]. Scores range from 0 to 40 with higher numbers indicating more symptoms (Ordinal Alpha = 0.8).

#### Alcohol use

Caregivers were asked the number of days that they drank alcohol in the last 30 days. Response categories ranged from 0 to 6 and the measure was treated as a continuous variable (0, 1–2, 3–5, 6–9, 10–19, 20–29, or all 30 days).

#### Relationship quality

Questions on feelings of family connectedness were adapted from commonly used scales in adolescent health behavior surveys [60]. Adolescents were asked the frequency with which they “feel like my parents/caregivers care about me”; “feel safe at home”; “belong at home”; and “like to spend time at home”. Scores ranged from 0 to 12 with higher numbers indicating more connectedness (Ordinal Alpha = 0.72). The nine-item Relationship Structure Questionnaire [61] assessed the quality of caregivers’ emotional attachment with their intimate partner, scores ranged from 0 to 27 with higher numbers indicating positive attachment (Ordinal Alpha = 0.9).

#### Attitudes towards violence

Caregivers were rated on their agreement with the following statements: Parents must be in control of children at all times; sometimes parents must hit children to make them listen; children who misbehave should be physically disciplined; children should often fear their parents; sometimes physically disciplining children is the only way to make them respect you; and sometimes parents must hit children to make them learn. The scale, developed for the Good School Study, ranged in scores from 0 to 18, with higher scores indicating less acceptance of violence (Ordinal Alpha = 0.8).

#### Violence

The WHO Women’s Health and Life Events Questionnaire assessed if caregivers have ever experienced (women) or perpetrated (men) any emotional, physical, or sexually violent behaviors in their current intimate relationship. Included in the questionnaire are seven different acts of violence, for example: “Slapped, pushed, or shoved you?”; “Hit you with a fist or object that could hurt you, kicked, dragged, or beat you up?”; and “Did they ever force you to have sex with them when you did not want to?” The International Society for the Prevention of Child Abuse and Neglect Screening Tool (ICAST) [62] examined caregiver’s perpetration or adolescents’ experiences of any physical or emotional violence against the adolescent in the dyad. In the questionnaire, participants were asked about their experiences (adolescents) or perpetration (caregivers) of eight acts of violence such as “Insulted them, or called them rude or hurtful names?”; “Twisted their arm or any other body part, slapped them, pushed them or thrown something at them?”; “Cut them with a sharp object or burnt them?” Since adolescent reports could not be attributed to the specific caregiver in the dyad, caregiver reports of perpetrating violence were used in the analyses.

#### Intervention

Describes whether adolescents were enrolled in an intervention or control school during the original cluster randomized controlled trial.

### Data analysis plan

Descriptive analysis included mean and standard deviation (SD) for continuous variables; count and proportion (%) for categorical variables; median and range for ordinal variables. Association between binary or nominal variables was tested with cross tab and χ^2^ test. Group comparison was performed using ANOVA and Kruskall-Wallis non-parametric test for continuous and ordinal variables, respectively. Analyses were performed with SPSS and Mplus 7.4 [53].

The main outcome of the study identifies 4 types of adolescent-caregiver dyads, using caregiver reports of ever any violence: (0) No violence [1]; IPV ‘only’ [2]; VAC ‘only’ [3]; both IPV-VAC. Given the gendered nature of violence against women (and that men reported IPV perpetration whereas women reported on IPV victimization) the sample was separated by caregiver sex. Two multinomial logistic regressions (one for female caregivers and one for male) were performed to test the difference between the dyads with no violence (0 - reference group) and the other three types of dyads [1–3]. This statistical approach identifies variables that discriminate between the reference group and the remaining dyads performing three pairwise comparisons (i.e. 0 vs.1; 0 vs. 2; and 0 vs. 3). We reported odds-ratio and confidence intervals (CIs). Estimates with *p* values ≤.05 were considered statistically significant, values ≤.10 were considered as statistical trends [63, 64].

We imputed missing data using Markov Chain Monte Carlo approach with *N* = 200 imputations (See Appendix B). Maximum Likelihood with Robust standard error (MLR) estimation was used, to account for potential outliers and non-normal distribution [53]. Multinomial logistic regressions were adjusted for intra-class (schools) correlation. Given that this study is a secondary analysis of trial data, no formal power calculations were carried out for this study. However, the main trial was powered to detect a difference of 13% in the primary outcome (past-week physical violence from school staff) with a 5% level of significance and 80% power [47, 52].

## Results

Tables 2 and 3 present descriptive and bivariate statistics of psychosocial covariates with the four violence categories (none, IPV ‘Only’, VAC ‘Only’, IPV and VAC), separated for male and female caregiver-adolescent dyads, respectively. The average age of adolescents range from 13.99 to 14.50 years for those in the male caregiver dyad and 13.85 to 13.99 years for those in the female caregiver dyads. The percentage of adolescents who reported living mostly with a biological mother ranged from 35.71 to 47.58% for those in the female caregiver dyads and 25.37 to 35.0% in the male caregiver dyads. Most adolescents did not report any physical disability (male caregiver-dyad range: 75.76 to 85.0%; female caregiver-dyad range: 69.57 to 85.56%). Caregivers’ average age was 40.9 (SD = 10.8). Most caregivers lived with their partner/spouse (90.7%) and were of Catholic or Christian religion (79.2%). A minority of caregivers, 9.76 to 35.48% of women and 19.61 to 33.33% of men, had attended secondary school or beyond. Caregivers reported being with their intimate partner for a median number of 17 years and most said that they drank 0 or only 1–2 days in the last month.

**Table 2:** Bivariate analysis of covariates with male caregiver-adolescent dyads

	None	IPV ‘Only’	VAC ‘Only’	IPV and VAC	*p*
	*n* = 66	*n* = 20	*n* = 67	*n* = 51	
Caregiver Report					
Age, mean (SD)	49.68 (14.42)	45.35 (9.78)	44.22 (11.27)	45.69 (9.62)	0.06
Education, % (n)					
*Primary*	68.75 (44)	66.67 (12)	73.85 (48)	80.39 (41)	0.49
*High School or more*	31.25 (20)	33.33 (6)	26.15 (17)	19.61 (10)	
Religion, % (n)					
*Catholic/Christian*	84.85 (56)	75 (15)	79.1 (53)	78.43 (40)	0.71
*Muslim*	15.15 (10)	25 (5)	20.9 (14)	21.57 (11)	
SES, mean (SD)	−0.06 (0.24)	0.06 (0.27)	0 (0.22)	0 (0.27)	0.28
Mental distress (SRQ), mean (SD)	3.97 (3.71)	5.45 (4.36)	5.07 (3.73)	6.43 (3.96)	0.01
Alcohol, median (min-max)^a^	0 (0–5)	0 (0–6)	0 (0–6)	0 (0–6)	0.10
Partner Living Status, % (n)					
*Do not live together*	6.06 (4)	5 (1)	4.48 (3)	1.96 (1)	0.76
*Live together*	93.94 (62)	95 (19)	95.52 (64)	98.04 (50)	
Intimate Partner Emotional Attachment, mean (SD)	15.38 (3.17)	14.68 (3.02)	14.66 (3.12)	13.86 (2.41)	0.07
Attitudes toward VAC, mean (SD)	12 (3.16)	14.25 (3.37)	12.66 (3.78)	15.39 (4.2)	0.00
Adolescent Report					
Sex, % (n)					
*Female*	40.91 (27)	50 (10)	61.19 (41)	43.14 (22)	0.09
*Male*	59.09 (39)	50 (10)	38.81 (26)	56.86 (29)	
Children age, mean (SD)	14.08 (1.21)	14.5 (1.28)	13.99 (1.24)	14.35 (0.98)	0.18
Physical disability, % (n)					
*No*	75.76 (50)	85 (17)	83.58 (56)	82.35 (42)	0.63
*Yes*	24.24 (16)	15 (3)	16.42 (11)	17.65 (9)	
Family Structure, % (n)					
*Lives with bio mother*	31.82 (21)	35 (7)	25.37 (17)	31.37 (16)	0.96
*Lives with bio father*	9.09 (6)	10 (2)	5.97 (4)	7.84 (4)	
*Lives with both bio parents*	27.27 (18)	25 (5)	38.81 (26)	31.37 (16)	
*Lives with neither bio parent*	31.82 (21)	30 (6)	29.85 (20)	29.41 (15)	
Mental health difficulties (SDQ), mean (SD)	14.58 (5.05)	14.91 (5.62)	14.88 (5.18)	14.28 (5.01)	0.711
Belong, mean (SD)	14.88 (4.8)	13.8 (3.19)	14.28 (4.68)	14.12 (4.87)	0.74
Study arm, % (n)					
*Control*	45.45 (30)	35.00 (7)	62.69 (42)	43.14 (22)	0.07
*Intervention*	54.55 (36)	65.00 (13)	37.31 (25)	56.86 (29)	

*ANOVA* for continuous variables; Chi-square for nominal variables*;* ^a^Kruskall-Wallis for ordinal variables

**Table 3:** Bivariate analysis of covariates with female caregiver-adolescent dyads

	None	IPV ‘Only’	VAC ‘Only’	IPV and VAC	*p*
	*n* = 71	*n* = 46	*n* = 90	*n* = 124	
Caregiver Report					
Age, mean (SD)	36.1 (9.7)	38.87 (10.37)	37.98 (8.05)	37.23 (6.48)	0.30
Education, % (n)					
*Primary*	64.52 (40)	90.24 (37)	76.19 (64)	88.79 (103)	< 0.01
*High School or more*	35.48 (22)	9.76 (4)	23.81 (20)	11.21 (13)	
Religion, % (n)					
*Catholic/Christian*	81.43 (57)	76.09 (35)	76.67 (69)	79.03 (98)	0.87
*Muslim*	18.57 (13)	23.91 (11)	23.33 (21)	20.97 (26)	
SES, mean (SD)	−0.01 (0.24)	−0.03 (0.3)	−0.1 (0.29)	−0.02 (0.27)	0.09
Mental distress (SRQ), mean (SD)	4.72 (3.62)	6.72 (4.64)	5.21 (3.93)	7.3 (4.07)	0.00
Alcohol, median (min-max) ^a^	0 (0–1)	0 (0–2)	0 (0–4)	0 (0–4)	0.07
Partner Living Status, % (n)					
*Do not live together*	14.08 (10)	4.35 (2)	13.33 (12)	13.71 (17)	0.36
*Live together*	85.92 (61)	95.65 (44)	86.67 (78)	86.29 (107)	
Intimate Partner Emotional Attachment, mean (SD)	14.87 (4.6)	17.37 (5.49)	15 (5.7)	18.56 (6.52)	0.00
Attitudes toward VAC, mean (SD)	15.01 (3.1)	14.19 (2.62)	14.08 (2.74)	13.74 (2.89)	0.04
Adolescent Report					
Sex, % (n)					
*Female*	45.07 (32)	50 (23)	36.67 (33)	37.9 (47)	0.36
*Male*	54.93 (39)	50 (23)	63.33 (57)	62.1 (77)	
Children age, mean (SD)	13.99 (1.18)	13.85 (1.17)	13.99 (1.24)	13.89 (1.24)	0.87
Physical disability, % (n)					
*No*	80.28 (57)	69.57 (32)	85.56 (77)	76.61 (95)	0.15
*Yes*	19.72 (14)	30.43 (14)	14.44 (13)	23.39 (29)	
Family Structure, % (n)					
*Lives with bio mother*	35.71 (25)	41.3 (19)	40 (36)	47.58 (59)	0.81
*Lives with bio father*	7.14 (5)	6.52 (3)	6.67 (6)	3.23 (4)	
*Lives with both bio parents*	25.71 (18)	19.57 (9)	24.44 (22)	25 (31)	
*Lives with neither bio parent*	31.43 (22)	32.61 (15)	28.89 (26)	24.19 (30)	
Mental health difficulties (SDQ), mean (SD)	14.3 (5.29)	15.39 (6.36)	15.32 (5.51)	14.35 (5.08)	0.42
Belong, mean (SD)	7.23 (2.1)	7.48 (2.22)	7.21 (2.31)	7.27 (2.16)	0.92
Study arm, % (n)					
*Control*	42.25 (30)	52.17 (24)	54.44 (49)	52.42 (65)	0.46
*Intervention*	57.75 (41)	47.83 (22)	45.56 (41)	47.58 (59)	

*ANOVA* for continuous variables; Chi-square for nominal variables; ^a^Kruskall-Wallis for ordinal variables

Bivariate analyses were conducted across four categories of adolescent-caregiver dyads (no violence, IPV ‘only’, VAC ‘only’, or IPV-VAC), separately for male and female caregiver-adolescent dyads. Significant differences were observed for the following variables for female caregiver-adolescent dyads: education (female caregivers with higher education or above were more likely to be in the ‘no violence’ dyad), mental distress (lower among female caregivers reporting no violence), and perception of closeness to one’s intimate partner (female caregiver-adolescent dyads with no violence were characterized with higher levels of closeness), and attitudes towards VAC (female caregiver-adolescent dyads with no violence reported more progressive attitudes). Significant demographic and/or psychosocial differences between violence categories observed for male caregiver-adolescent dyads included: age (older male caregivers were more likely to report no violence); mental distress (less among male caregivers in the dyads reporting no violence); and attitudes towards VAC (male caregiver reporting no violence also reported more progressive attitudes).

Table 4 presents the percentage overlap of VAC and IPV among the adolescent-caregiver dyads, separated by sex of the caregiver. Over one third (37.46%) of female caregiver-adolescent dyads reported both VAC and IPV, compared to 25.0% of male caregiver-adolescent dyads. In contrast, 32.4% of men caregivers reported no violence, compared to 21.45% of women caregivers. The combined totals for both male and female caregiver dyads found 33% of dyads reported both IPV and VAC, 29% reported VAC “only”, 12% reported IPV “only”, and 26% reported neither VAC or IPV. Overall, dyads reporting any IPV were more likely to report any VAC (χ^2^ = 14.9, df = 1, *p* < .001). This association remained significant on separate analyses of reports from women and male caregiver.

**Table 4:** Percentage of dyads reporting none, IPV ‘Only’, VAC ‘Only’, or IPV and VAC, by sex of caregiver

	None	IPV ‘Only’	VAC ‘Only’	IPV and VAC
Women	21.45% (*n* = 71)	13.9% (*n* = 46)	27.19% (*n* = 90)	37.46% (*n* = 124)
Men	32.4% (*n* = 66)	9.8% (*n* = 20)	32.8% (*n* = 67)	25.0% (*n* = 51)

Chi-square *=* 14.9*, p =* 0.0007*;* adjusted for clustering: schools

In order to explore associations between risk factors and reporting of IPV, VAC, or both, we ran separate multinomial logistic regression models for female and male caregivers. Below we summarize the associations that were significant (or approaching significance), comparing dyads reporting either IPV and VAC, IPV ‘Only’, or VAC ‘Only’ to those with no reported violence (Table 5). The results for dyads with female caregivers showed that those with IPV-VAC were characterized as caregivers with less education, lower SES, more mental distress, more frequent alcohol use, slightly longer relationship duration (approaching significance), and less emotional attachment to their intimate partner. Dyads of male caregivers with IPV-VAC were characterized as having more frequent caregiver alcohol use (approaching significance),

**Table 5:** Multinomial logistic regressions of IPV and VAC, VAC only, and IPV only, compared to no violence dyads

	Female Caregivers			Male Caregivers		
Odd-ratio (CI)	IPV and VAC (*n* = 124)	VAC ‘only’ (*n* = 90)	IPV ‘only’ (*n* = 46)	IPV and VAC (*n* = 51)	VAC ‘only’ (*n* = 67)	IPV ‘only’ (*n* = 20)
Caregiver Reports						
Age (years)	0.96 (0.9–1.01)	1.02 (0.97–1.08)	0.99 (0.92–1.05)	0.96 (0.91–1.02)	0.95 (0.91–1)^*^	1(0.95–1.05)
Education (some secondary or higher vs. primary or no)	0.23 (0.09–0.59)^**^	0.52 (0.21–1.26)	0.3 (0.09–0.98)^*^	0.87 (0.29–2.61)	0.97 (0.37–2.51)	0.9 (0.25–3.29)
Religious status^a^	1.42 (0.61–3.32)	1.21 (0.52–2.86)	1.43 (0.53–3.86)	1.62 (0.51–5.1)	1.7 (0.6–4.85)	3.22 (0.86–12.18)^¥^
SES	0.26 (0.07–1.01)^*^	0.13 (0.03–0.51) ^**^	0.31 (0.06–1.55)	1.52 (0.23–10.07)	3.39 (0.61–18.73)	4.44 (0.46–42.52)
Mental Distress (SRQ)	1.12 (1.01–1.22)^*^	1.02 (0.92–1.13)	1.07 (0.97–1.2)	1.09(0.97–1.23)	1.07 (0.96–1.2)	1.04 (0.9–1.2)
Alcohol Use	3.03 (0.99–9.3)^*^	2.59 (0.84–8)^¥^	1.82 (0.48–6.96)	1.26 (0.95–1.67)^¥^	1.09 (0.83–1.43)	1.38 (0.99–1.93)^¥^
Intimate Partner Current Living Status (living together vs. not)	1.09 (0.37–3.22)	0.92 (0.31–2.77)	2.39 (0.5–11.36)	6.42 (0.46–90.02)	4.1 (0.55–30.27)	3.97 (0.39–40.04)
Intimate Partner Relationship Length (years)	1.05 (0.99–1.11) ^¥^	1.01 (0.95–1.06)	1.04 (0.98–1.12)	1.03 (0.97–1.09)	1.01 (0.95–1.06)	0.94 (0.87–1.01)^¥^
Intimate Partner Emotional Attachment	0.9 (0.85–0.97)^**^	0.99 (0.93–1.06)	0.92 (0.86–1)^**^	0.8 (0.71–0.91)^***^	0.96 (0.86–1.08)	0.84 (0.73–0.98)^*^
Attitudes against VAC	0.95 (0.84–1.07)	0.92 (0.82–1.05)	0.94 (0.81–1.09)	0.86 (0.73–1)^*^	0.94 (0.82–1.07)	0.96 (0.79–1.15)
Adolescent Reports						
Age (years)	0.95 (0.73–1.25)	1.02 (0.77–1.35)	0.92 (0.67–1.28)	1.27 (0.86–1.88)	0.86 (0.61–1.22)	1.39 (0.9–2.18)
Sex of child (girls vs. boys)	1.14 (0.58–2.23)	1.32 (0.66–2.61)	0.76 (0.34–1.65)	1.11 (0.45–2.72)	0.39 (0.17–0.87)^*^	0.93 (0.31–2.77)
Mental Distress (SDQ)	0.98 (0.92–1.04)	1.04 (0.98–1.11)	1.02 (0.95–1.09)	0.95 (0.87–1.05)	0.98 (0.9–1.07)	0.95 (0.84–1.06)
Physical Disability	0.97 (0.44–2.18)	0.63 (0.26–1.51)	1.35 (0.54–3.39)	0.63 (0.25–1.52)	0.55 (0.24–1.26)	0.76 (0.26–2.2)
Lives with at least one biological parent	1.68 (0.79–3.53)	1.52 (0.7–3.32)	1.84 (0.76–4.48)	0.93 (0.34–2.51)	1.23 (0.49–3.13)	1.75 (0.49–6.23)
Lives with both biological parents	0.99 (0.39–2.51)	1.48 (0.59–3.67)	1.06 (0.35–3.25)	0.9 (0.25–3.22)	2.34 (0.8–6.75)	3.35 (0.75–14.88)
Child sense of belonging and safety at home	1 (0.86–1.16)	0.98 (0.84–1.14)	1.06 (0.89–1.27)	0.98 (0.79–1.22)	1.01 (0.81–1.26)	1.28 (0.99–1.67)^¥^
Intervention	0.63 (0.32–1.23)	0.76 (0.38–1.51)	0.61 (0.27–1.38)	1.4 (0.57–3.42)	0.61 (0.27–1.34)	1.75 (0.58–5.21)

^a^Muslim affiliation compared to Christian/Catholic (ref); ^b^ Compared to no biological parents; ^¥^ *p* < 0.10; ^*^ *p* < 0.05; ^**^ *p* < 0.01; ^***^ *p* < 0.001

less emotional attachment to their intimate partner, and attitudes more accepting of using violence against children as discipline.

Dyads with female caregivers with VAC ‘only’ were more likely to have lower SES and more frequent caregiver alcohol use (approaching significance). The male caregiver dyads with VAC ‘only’ were characterized as having younger caregivers and adolescent boys (rather than girls).

Dyads of female caregivers with ‘only’ IPV were associated with lower education status and less reported emotional attachment to their intimate partner. Male caregivers with ‘only’ IPV reported having a Muslim religious affiliation (approaching significance), more alcohol use (approaching significance), shorter relationship duration (approaching significance), less emotional attachment to their partner, and adolescents reporting increased connectedness in the family (approaching significance).

## Discussion

This exploratory study is one of the first in any low- or middle-income country (LMIC) to investigate the overlap and contributing factors of intimate partner violence against women (IPV) and violence against children (VAC) using a sample of adolescents and their caregivers in Uganda. Similar to previous research on the intersection of IPV and VAC from high income countries [11, 14, 16], caregivers who reported perpetrating (men) or experiencing (women) IPV were also more likely to report perpetrating VAC. Over one third (37.46%) of women reported experiencing IPV and using violence against children and 21.30% of men reported perpetrating both IPV and VAC. The 33% rate of overlap between IPV and VAC is markedly higher than other studies primarily from the United States, where probability samples have found rates of 6–12% [65]. Overlap in the current study only captures violence experienced or perpetrated by the specific caregiver and adolescent interviewed; household rates that include violence from other caregivers or family members are likely to be higher. This evidence supports calls for violence researchers to begin “recognizing that co-occurrence is the norm, not the exception” [7], which may be particularly true in settings with overall higher rates of multiple forms of violence.

Results highlight the unique patterns of IPV and VAC in the family depending on caregiver sex. Consistent with IPV literature [66], female caregiver education and socioeconomic status were found to be protective factors for women from both experiencing IPV and using VAC. Secondary education may be associated with attitudes rejecting IPV (for which a measure was not included), and for women, increased employment and time outside the home. Limited resources or upward mobility, as evident from the SES results, may uniquely affect women’s likelihood to experience violence from her partner and/or navigate stress through the use of violence against children—consistent with other research indicating that economic marginalization and/or dependence on men compromises a women’s ability to exit violent relationships [67].

Sex of the child was the only statistically significant adolescent factors predicting risk of violence (family connectedness was approaching significance in the male caregiver IPV ‘only’ dyad). Results indicate that compared to girls, boys are at a higher risk of experiencing violence by male caregivers in the VAC ‘only’ dyad. This finding may reflect that there is greater social acceptance of men’s use of violence against boys compared to girls.

Female caregivers who both experience IPV and use VAC (the IPV and VAC dyads) reported more mental distress, compared to those neither experiencing or perpetrating violence (no violence dyads). Women’s mental distress was not associated with experiencing IPV ‘only’ or perpetrating VAC ‘only’, suggesting there is added distress for women associated with being in a family with both IPV and VAC. A recent qualitative study in Uganda exploring patterns of intersecting IPV and VAC revealed how—in the context of patriarchal families and male authority—women who are experiencing IPV may at times use violence against their children as a way to redirect or transfer their own trauma and powerlessness vis-à-vis their husbands [37]. Given the link between poor mental health and violence in the IPV literature [3, 44], further research is needed to understand if this finding holds in other contexts. Neither adolescents’ nor men’s reports of mental health was associated with an increased risk of violence in any of the dyads.

While alcohol use is a risk factor for men’s use of IPV [68] and caregiver use of VAC [69], this study is one of the first to examine the association of alcohol and co-occurring IPV and VAC. Female caregiver reports of more frequent alcohol use were significantly associated with their likelihood of being in the IPV and VAC dyads, and was approaching significance in the IPV ‘only’ dyad. When considered alongside the finding related to women’s increased mental distress (reported above), women’s alcohol use may be a coping strategy for their broader experiences of violence and powerlessness in the family [37], which at the same time exacerbates their own use of violence against children. Approaching significance, male caregivers in the IPV ‘only’ dyad and the IPV and VAC dyads also reported more frequent alcohol use. Qualitative research has found men’s alcohol use to be a compounding factor for violence in the family, as well as a proximate trigger for men’s use of both IPV and VAC [37]. Overall associations between violence and alcohol is among the most consistent result in the analysis, underscoring that more research is needed to tease out the dynamics between caregivers’ alcohol use in families with co-occurring IPV and VAC, including female caregivers’ alcohol use.

Stronger perceived emotional attachment between intimate partners consistently emerged as a protective factor for the IPV-VAC dyads and IPV ‘only’ dyads for both male and female caregivers. This emphasis on the quality of intimate relationships has also been raised in previous research from Uganda, for example in explaining processes of change towards healthier relationship dynamics between couples previously experiencing violence [70]. Of particular interest is the inverse association between higher intimate partner emotional attachment and dyads with both IPV and VAC. Perhaps women and men with stronger emotional attachment are less likely to experience/perpetrate IPV, which also reduces their conflict over parenting and their use of VAC. Alternatively, partners who practice more positive parenting and less violence may have less conflict in their intimate relationships and subsequently feel more positive attachment to their partner. Regardless, these findings emphasize that family relationships are interdependent, whereby the perceived quality of the relationship between adults also affects children in the home.

Men with attitudes rejecting VAC were less likely to report using both IPV and VAC. This aligns with expectations that lower acceptance of violence correlates with less violent behaviors [71]. While the survey did not include attitudinal questions about IPV, future research should investigate whether or not attitudes rejecting IPV may also result in reduced VAC perpetration.

This study had some limitations. Caregivers were recruited through the adolescents, resulting in a lower caregiver response rate. The sample of adolescents whose caregivers participated in the study (although similar in age, sex, and SES) reported better attendance in the past week, perhaps biasing the dyad sample (See Appendix C). Caregiver reports of perpetrating VAC against the specific adolescent in the sample were used, because adolescents were asked about perpetration from *any* caregiver. Perpetration of VAC may have been under-reported by caregivers, although violence for purposes of disciplining children is widely accepted in Uganda and unlikely to be significantly under-reported [32]. Using caregiver reports may have had other impact on results, such as a non-significant association with child mental health. Both primary and non-primary caregivers completed the survey, perhaps altering the results and underestimating the children’s exposure to violence if only primary caregivers had been interviewed. Data came from a school-based sample and results may differ from dyads with an adolescent not in school. In addition, the primary Good School Study was not explicitly designed for these analyses, and subsequently some of the sub-groups have a small sample size (notably male caregiver-adolescent dyads in the ‘IPV-only’ group), potentially limiting statistical power. Finally, given the cross-sectional nature of the data, we cannot draw conclusions about hypothesized causal relationships between variables. For example, causal ordering is particularly ambiguous for the relationship between intimate partner violence and intimate partner living status or emotional attachment with an intimate partner.

## Conclusions

Results from this study suggest several implications for violence prevention and response programming and research. While VAC and IPV are affecting the majority of families/dyads, one third of dyads are reporting both types of violence concurrently. Efforts to integrate prevention programming may consider addressing the associated factors, especially in families characterized by both IPV and VAC (i.e., women’s education, socio-economic status, alcohol use, and emotional attachment between intimate partners). Furthermore, almost all factors associated with either the VAC ‘only’ or IPV ‘only’ dyads, were also associated with the IPV and VAC dyads. More evaluative research is needed to better understand the potential ‘spillover effects’ of violence prevention interventions targeting either VAC or IPV on the prevention of other types of violence. For example, VAC prevention programming (especially ones that focus on poorer communities, or programs which may focus on preventing violence against girls) should also measure their potential effects on preventing IPV. Likewise, IPV prevention programming that focuses on improving women’s education or intimate partner relationships should also evaluate potential outcomes on VAC.

## Data Availability

The data are being collected as part of a project with multiple partners, and the authors have a donor agreement that all data will remain within the project until being made available via a controlled access procedure in 2024. The datasets generated and/or analyzed during the current study will be publicly available on The London School of Hygiene & Tropical Medicine repository at that time.

## Appendix A

**Table 6:** Socioeconomic Status Items and Results of Confirmatory Factor Analysis

Item	Scale	Loading	
		Initial results	After excluding non-sig. Items
Do you own or rent a property, live somewhere without paying, or does your employer pay for your accommodation?	1 = Does not own 2 = Owns	.31	.36
How many children aged 17 years and younger sleep in the same sleeping area with you?	0 = No children 1 = One child 2 = More than one child	.27	.25
Thinking about your house where you live now, what is the main material of the floor	1 = Earth, sand, or dung 2 = Ceramic, cement, carpet	.68	.70
Does your household own electricity?	1 = No 2 = Yes	.92	.93
Does your household own a radio?	1 = No 2 = Yes	.34	.31
Does your household own a television?	1 = No 2 = Yes	.97	.97
Does any member of your household own a watch?	1 = No 2 = Yes	.38	.37
Does any member of your household own a mobile phone?	1 = No 2 = Yes	.49	.44
Does any member of your household own a motorcycle?	1 = No 2 = Yes	.29	.26
Does any member of your household own land that can be used for agriculture? ^¥^	1 = No 2 = Yes	.16	–
Does any member of your household own livestock, herds, other farm animals, or poultry? ^¥^	1 = No 2 = Yes	.11	–
Does any member of your household own a bicycle? ^¥^	1 = No 2 = Yes	.06	
Yesterday, how many meals did you eat? (Ate at least two meals yesterday) ^¥^	0 = No or one meal 1 = Two or more meals	.10	–
How many adults aged 18 years and older sleep in the same sleeping area with you?^¥^	0 = No adults 1 = One adult 2 = More than one adult	.07	–

^¥^dropped because of non-significant *p* value (>.05)

## Appendix B

**Table 7:** Missing Data

		Women		Men
	n	%	n	%
VAC-IPV	331	0.00	204	0.00
Education	303	8.46	198	2.94
Relationship Status	331	0.00	204	0.00
Religion	330	0.30	204	0.00
Family structure	330	0.30	204	0.00
Child Disability	331	0.00	204	0.00
VAC attitudes	308	6.95	193	5.39
CHILD Belong	331	0.00	203	0.49
Intimate Partner Emotional Attachment	331	0.00	204	0.00
Child mental health (SDQ)	331	0.00	204	0.00
Caregiver mental health (SRQ)	331	0.00	204	0.00
Adolescent age	330	0.30	204	0.00
Caregiver age	331	0.00	204	0.00
Child sex	331	0.00	204	0.00
Alcohol	331	0.00	202	0.98
SES	331	0.00	204	0.00

## Appendix C

**Table 8:** Demographic characteristics of full adolescent sample from class P7, compared to adolescent sample from class P7 whose caregivers participated in survey (Note: all P7 students’ caregivers were invited to participate)

	Full adolescent P7 sample (*n* = 1171)	Adolescent P7 sample with matched caregivers (*n* = 800)
Age, mean (SD)	13.95 (1.3)	13.99 (1.2)
Sex, % (n)		
*Female*	54.57 (639)	56.00 (448)
*Male*	45.43 (532)	44.00 (352)
Past week school absences, % (n)		
*No days missed*	85.89 (998)	82.84 (657)
*One or more days missed*	14.11 (164)	17.16 (140)
Meals eaten yesterday		
*1 meal or fewer*	13.25 (155)	15.18 (121)
*2 meals*	38.89 (455)	40.78 (325)
*3 or more meals*	47.86 (560)	44.04 (351)

## Abbreviations

ANOVA: Analysis of variance
CFA: Confirmatory factor analysis
CFI: Comparative fit index
DF: Degrees of freedom
GST: Good School Toolkit
IPSCAN: International Society for the Prevention of Child Abuse and Neglect Screening Tool
IPV: Intimate partner violence against women
LMIC: Low- or middle-income country
MLR: Maximum Likelihood with Robust standard error
P: *P*-value
RMSEA: Root mean square error of approximation
SD: Standard deviation
SDQ: Strengths and difficulties questionnaire
SES: Socioeconomic status
SPSS: **S**tatistical Package for Social Sciences
SRQ: Self-report questionnaire
TLI: Tucker-Lewis index
VAC: Violence against children
WHO: World Health Organization
WLSMV: Weighted Least Square with adjusted Mean and Variance

## References

[1] Devries KM, Mak JY, Garcia-Moreno C, Petzold M, Child JC, Falder G (2013). Global health. The global prevalence of intimate partner violence against women. Science..

[2] Hillis S, Mercy J, Amobi A, Kress H (2016). Global prevalence of past-year violence against children: a systematic review and minimum estimates. Pediatrics..

[3] Ellsberg M, Jansen HAFM, Heise L, Watts CH, Garcia-Moreno C (2008). Intimate partner violence and women's physical and mental health in the WHO multi-country study on women's health and domestic violence: an observational study. Lancet.

[4] Norman RE, Byambaa M, De R, Butchart A, Scott J, Vos T (2012). The long-term health consequences of child physical abuse, emotional abuse, and neglect: a systematic review and meta-analysis. PLoS Med.

[5] Guedes A, Bott S, Garcia-Moreno C, Colombini M (2016). Bridging the gaps: a global review of intersections of violence against women and violence against children.

[6] Fry DA, Elliott SP (2017). Understanding the linkages between violence against women and violence against children. Lancet Glob Health.

[7] Hamby S, Grych J (2013). The web of violence: exploring connections among different forms of interpersonal violence and abuse.

[8] Maternowska C, Shakel R, Carlson C, Levtov R, Heise L. The global politics of the age-gender divide: Violence against women and violence against children. under review. 10.1080/17441692.2020.1805783 32835618

[9] Moore JG (1975). Yo-yo children—victims of matrimonial violence. Child welfare.

[10] Levine MB (1975). Interparental violence and its effect on the children: a study of 50 families in general practice. Med Sci Law.

[11] Appel AE, Holden GW (1998). The co-occurrence of spouse and physical child abuse: a review and appraisal. J Fam Psychol.

[12] Edleson JL (1999). The overlap between Child maltreatment and woman battering. Violence Against Women.

[13] Edleson JL (1995). Do batterers’ programs work. University of Minnesota and Domestic Abuse Project Inc, paper presented at the International Study Group on the Future of Intervention with Battered Women and Their Families, Israel.

[14] Hamby S, Finkelhor D, Turner H, Ormrod R (2010). The overlap of witnessing partner violence with child maltreatment and other victimizations in a nationally representative survey of youth. Child Abuse Negl.

[15] Casanueva C, Martin SL, Runyan DK (2009). Repeated reports for child maltreatment among intimate partner violence victims: findings from the National Survey of Child and adolescent well-being. Child Abuse Negl.

[16] Hartley CC (2002). The co-occurrence of Child maltreatment and domestic violence: examining both neglect and Child physical abuse. Child Maltreatment.

[17] Bourassa C (2007). Co-occurrence of Interparental violence and Child physical abuse and It’s effect on the adolescents’ behavior. J Fam Violence.

[18] Mbilinyi LF, Edleson JL, Hagemeister AK, Beeman SK (2007). What happens to children when their mothers are battered? Results from a four City anonymous telephone survey. J Fam Violence.

[19] Tajima EA (2004). Correlates of the co-occurrence of wife abuse and child abuse among a representative sample. J Fam Violence.

[20] Damant D, Lapierre S, Lebossé C, Thibault S, Gv L, Hamelin-Brabant L (2010). Women’s abuse of their children in the context on domestic violence: reflection from women’s accounts. Child Fam Soc Work.

[21] Beeman SK, Hagemeister AK, Edleson JL (2001). Case assessment and service receipt in families experiencing both Child maltreatment and woman battering. J Int Violence.

[22] O’Keefe M (1994). Linking marital violence, mother-child/father-child aggression, and child behavior problems. J Fam Violence.

[23] Ross SM (1996). Risk of physical abuse to children of spouse abusing parents. Child Abuse Negl.

[24] Gracia E, López-Quílez A, Marco M, Lila M (2018). Neighborhood characteristics and violence behind closed doors: the spatial overlap of child maltreatment and intimate partner violence. PLoS One.

[25] Wherry Jeffrey N., Medford Elizabeth A., Corson Kimberly (2015). Symptomatology of Children Exposed to Domestic Violence. Journal of Child & Adolescent Trauma.

[26] Moylan CA, Herrenkohl TI, Sousa C, Tajima EA, Herrenkohl RC, Russo MJ (2010). The effects of Child abuse and exposure to domestic violence on adolescent internalizing and externalizing behavior problems. J Fam Violence.

[27] Maniglio R (2009). The impact of child sexual abuse on health: a systematic review of reviews. Clin Psychol Rev.

[28] Fergusson DM, Boden JM, Horwood LJ (2008). Exposure to childhood sexual and physical abuse and adjustment in early adulthood. Child Abuse Negl.

[29] MacMillan HL, Fleming JE, Streiner DL, Lin E, Boyle MH, Jamieson E (2001). Childhood abuse and lifetime psychopathology in a community sample. Am J Psychiatr.

[30] Grethel SM (2004). Correlates of psychological symptoms among children exposed to domestic violence: severity of domestic violence exposure, child abuse, and psychosocial stressors: Pepperdine University.

[31] Fieggen A, Wiemann M, Brown C, Van As A, Swingler G, Peter J (2004). Inhuman shields-children caught in the crossfire of domestic violence: original article. S Afr Med J.

[32] Ayinmode B, Tunde-Ayinmode M (2009). Family violence among mothers seen at the University of Ilorin Teaching Hospital, Ilorin. Niger S Afr J Psychiatry.

[33] Saile R, Ertl V, Neuner F, Catani C (2014). Does war contribute to family violence against children? Findings from a two-generational multi-informant study in northern Uganda. Child Abuse Negl.

[34] HEISE LL (1998). Violence against women:an integrated. Ecol Framework Violence Against Women.

[35] Belsky J (1980). Child maltreatment: an ecological integration. Am Psychol.

[36] Gracia E, Rodriguez CM, Martín-Fernández M, Lila M. Acceptability of family violence: underlying ties between intimate partner violence and Child abuse. J Int Violence. 2017; 10.1177/0886260517707310. 10.1177/0886260517707310 29294751

[37] Namy S, Carlson C, O'Hara K, Nakuti J, Bukuluki P, Lwanyaaga J (2017). Towards a feminist understanding of intersecting violence against women and children in the family. Soc Sci Med.

[38] Dekel Bianca, Abrahams Naeemah, Andipatin Michelle (2018). Exploring the Intersection Between Violence Against Women and Children from the Perspective of Parents Convicted of Child Homicide. Journal of Family Violence.

[39] Heise L (2011). What works to prevent partner violence? An evidence overview.

[40] WHO/ISPCAN (2006). Preventing child maltreatment: A guide to taking action and generating evidence.

[41] Heise LL, Kotsadam A (2015). Cross-national and multilevel correlates of partner violence: an analysis of data from population-based surveys. Lancet Glob Health.

[42] Machisa MT, Christofides N, Jewkes R (2016). Structural pathways between Child abuse, poor mental health outcomes and male-perpetrated intimate partner violence (IPV). PLoS One.

[43] Oram S, Trevillion K, Khalifeh H, Feder G, Howard L. Systematic review and meta-analysis of psychiatric disorder and the perpetration of partner violence2013. 1–16 p. 10.1017/S2045796013000450 PMC7192171 23962668

[44] Devries KM, Mak JY, Bacchus LJ, Child JC, Falder G, Petzold M (2013). Intimate partner violence and incident depressive symptoms and suicide attempts: a systematic review of longitudinal studies. PLoS Med.

[45] Reading R, Bissell S, Goldhagen J, Harwin J, Masson J, Moynihan S (2009). Promotion of children's rights and prevention of child maltreatment. Lancet.

[46] Stith SM, Liu T, Davies LC, Boykin EL, Alder MC, Harris JM (2009). Risk factors in child maltreatment: a meta-analytic review of the literature. Aggress Violent Behav.

[47] Devries KM, Allen E, Child JC, Walakira E, Parkes J, Elbourne D (2013). The good schools toolkit to prevent violence against children in Ugandan primary schools: study protocol for a cluster randomised controlled trial. Trials..

[48] Devries Karen M, Knight Louise, Child Jennifer C, Mirembe Angel, Nakuti Janet, Jones Rebecca, Sturgess Joanna, Allen Elizabeth, Kyegombe Nambusi, Parkes Jenny, Walakira Eddy, Elbourne Diana, Watts Charlotte, Naker Dipak (2015). The Good School Toolkit for reducing physical violence from school staff to primary school students: a cluster-randomised controlled trial in Uganda. The Lancet Global Health.

[49] Devries KM, Child JC, Elbourne D, Naker D, Heise L (2015). “I never expected that it would happen, coming to ask me such questions”:ethical aspects of asking children about violence in resource poor settings. Trials..

[50] Ellsberg M, Heise L (2005). Researching violence against women: a practical guide for researchers and activists.

[51] Merg C (2012). Ethical principles, dilemmas and risks in collecting data on violence against children: a review of available literature.

[52] Devries KM, Child JC, Allen E, Walakira E, Parkes J, Naker D (2014). School violence, mental health, and educational performance in Uganda. Pediatrics..

[53] Muthén LK, Muthén BO (1998). Mplus User's Gude.

[54] Bentler PM (1990). Comparative fit indexes in structural models. Psychol Bull.

[55] Hu L-T, Bentler P, Hoyle RH (1995). Evaluating model fit. Structural equation modeling concepts, issues, and applications.

[56] Hu L, Bentler PM (1999). Cutoff criteria for fit indexes in covariance structure analysis: conventional criteria versus new alternatives. Struct Equ Model Multidiscip J.

[57] Zumbo BD, Gadermann AM, Zeisser C (2007). Ordinal versions of coefficients alpha and Theta for Likert rating scales. J Mod Appl Stat Methods.

[58] Beusenberg M, Orley J (1994). A User’s guide to the self reporting questionnaire (SRQ).

[59] Goodman R (2001). Psychometric properties of the strengths and difficulties questionnaire. J Am Acad Child Adolesc Psychiatry.

[60] McQueen MB, Boardman JD, Domingue BW, Smolen A, Tabor J, Killeya-Jones L (2015). The National Longitudinal Study of adolescent to adult health (add health) sibling pairs genome-wide data. Behav Genet.

[61] Fraley CR, Heffernan ME, Vicary AM, Brumbaugh CC (2011). The experiences in close relationships-relationship structures questionnaire: a method for assessing attachment orientations across relationships. Psychol Assess.

[62] Zolotor AJ, Runyan DK, Dunne MP, Jain D, Peturs HR, Ramirez C (2009). ISPCAN Child abuse screening tool Children's version (ICAST-C): instrument development and multi-national pilot testing. Child Abuse Negl.

[63] Thiese MS, Ronna B, Ott U (2016). P value interpretations and considerations. J thorac Dis.

[64] Namy S, Carlson C, Norcini Pala A, Faris D, Knight L, Allen E (2017). Gender, violence and resilience among Ugandan adolescents. Child Abuse Negl.

[65] Margolin G, Gordis EB (2003). Co-occurrence between marital aggression and parents' child abuse potential: the impact of cumulative stress. Violence Vict.

[66] Vyas S, Watts C (2009). How does economic empowerment affect women’s risk of intimate partner violence in low and middle income countries? A systematic review of published evidence. J Int Dev.

[67] Abramsky T, Watts CH, Garcia-Moreno C, Devries K, Kiss L, Ellsberg M (2011). What factors are associated with recent intimate partner violence? Findings from the WHO multi-country study on women's health and domestic violence. BMC Public Health.

[68] Foran HM, O’Leary KD (2008). Alcohol and intimate partner violence: a meta-analytic review. Clin Psychol Rev.

[69] Freisthler B, Needell B, Gruenewald PJ (2005). Is the physical availability of alcohol and illicit drugs related to neighborhood rates of child maltreatment?. Child Abuse Negl.

[70] Starmann E, Collumbien M, Kyegombe N, Devries K, Michau L, Musuya T (2017). Exploring Couples’ processes of change in the context of SASA!, a violence against women and HIV prevention intervention in Uganda. Prev Sci.

[71] Speizer IS (2009). Intimate partner violence attitudes and experience among women and men in Uganda. J Int Violence.

